# Functional characterization of MFSD3 in auditory system and zebrafish embryogenesis

**DOI:** 10.3389/fgene.2025.1634493

**Published:** 2025-09-15

**Authors:** Ying Ma, Shi-Wei Qiu, Wei-Qian Wang, Hai-Feng Feng, Lu Zheng, Ge-Ge Wei, Hui-Yi Nie, Jin-Yuan Yang, Yi-Jin Chen, Pu Dai, Xue Gao, Yong-Yi Yuan

**Affiliations:** ^1^ Senior Department of Otolaryngology Head and Neck Surgery, The 6th Medical Center of Chinese PLA General Hospital, Chinese PLA Medical School, Beijing, China; ^2^ State Key Laboratory of Hearing and Balance Science, Beijing, China; ^3^ National Clinical Research Center for Otolaryngologic Diseases, Beijing, China; ^4^ Key Laboratory of Hearing Science, Ministry of Education, Beijing, China; ^5^ Beijing Key Laboratory of Hearing Impairment Prevention and Treatment, Beijing, China; ^6^ School of Life Sciences, Tsinghua University, Beijing, China; ^7^ Chinese Institute for Brain Research, Beijing, China; ^8^ Department of Otolaryngology, PLA Rocket Force Characteristic Medical Center, Beijing, China

**Keywords:** MFSD3, morpholino knockdown, zebrafish, auditory system, wnt/β-catenin

## Abstract

The solute carriers (SLCs) are important membrane-bound transporters that regulate cellular nutrition, metabolism, homeostasis and survival. Emerging evidence highlights the critical involvement of SLCs in auditory physiology. To date, over ten SLC family members have been linked to hearing function. MFSD3 (also known as SLC33A2), is a putative plasma membrane-localized acetyl-CoA transporter regulating lipid metabolism and energy homeostasis. It has been found to be associated with the pathogenesis of neurodegenerative dementia and tumor progression. Nevertheless, its potential role in hearing remains unexplored. In this study, through qRT-PCR, we demonstrated that *mfsd3* was predominantly expressed during early embryonic development in zebrafish. Morpholino-mediated *mfsd3* knockdown in zebrafish induced inner ear malformations (hypoplastic otic vesicles, reduced otolith size) and hair cells loss in lateral line neuromasts. Additionally, Mfsd3 deficiency led to developmental defects (pericardial edema, body axis curvature) and impaired locomotor activity in zebrafish. The qRT-PCR analysis further revealed significant upregulation of key Wnt/β-catenin pathway components (*dkk1b*, *wnt8a*, *lrp6*, *frzb* and *COX2*) in *mfsd3* knockdown zebrafish. Our findings suggest *MFSD3* as a potential participant in auditory function and embryogenesis, with implications for understanding hearing loss pathogenesis.

## 1 Introduction

Hearing loss (HL) affects over 1.5 billion people worldwide, with profound impacts on speech development, communication, cognition, education and mental health (Chadha et al., 2021). Genetic factors play a major role, as evidenced by a recent mouse study identifying over 1,000 genes potentially involved in hearing maintenance (Ingham et al., 2019). Given this complexity, the discovery of novel hearing-related genes and their functions remains critical.

Membrane transporters, particularly solute carriers (SLCs) proteins, are physiologically important for cellular homeostasis and survival (Hediger et al., 2013). Over ten SLCs genes have been found to be associated with hearing impairment, including *SLC26A4* (MIM *605646), *SLC44A4* (MIM *606107), *SLC7A14* (MIM *615720), *SLC12A2* (MIM *600840), *SLC17A8* (MIM *607557) and *SLC22A4* (MIM *604190) (Qian et al., 2022). The major facilitator superfamily domain containing protein 3 (MFSD3) encoded by *MFSD3* (MIM *620308, NM_138431) gene, also known as SLC33A2, belongs to the SLC superfamily and is predicted to function as an acetyl-CoA transporter localized in the plasma membrane (Perland et al., 2017a; Perland et al., 2017b). Heterozygous variants in *MFSD3* have been associated with dementia with Lewy bodies (MIM #127750), while its overexpression has been linked to metastatic progression in uveal melanoma (MIM #155720) (Li et al., 2019; Shigemizu et al., 2022). However, the role of *MFSD3* in hearing has not been explored. We previously identified a missense variant in *MFSD3* that co-segregated with HL in a Chinese Han family using whole-genome sequencing (Supplementary Figure S1). According to the HL-specific ACMG/AMP guidelines (Oza et al., 2018), this variant was classified as a variant of uncertain significance (VUS). And no variant in the *MFSD3* gene has been detected in other known deafness families. Therefore, it is essential to investigate the potential association between *MFSD3* and HL through functional experiments.

The zebrafish is an ideal model for hearing research due to its transparent body, rapid development, genetic tractability and conserved orthologs of human deafness genes (Vona et al., 2020). They possess two mechanosensory systems, the inner ear and the lateral line. The inner ear shares structural and functional similarities with that of mammals and is essential for auditory and vestibular functions. In contrast, the lateral line system detects local water motion and facilitates behaviors including prey detection, predator avoidance and social interactions. Anatomically, the lateral line is organized into two components: the cranial lateral line (anterior lateral line) consisting of the head neuromasts, and the trunk lateral line (posterior lateral line), which is composed of a continuous canal housing multiple neuromasts. The neuromast contains a superficial cluster of hair cells surrounded by supporting cells. These lateral line hair cells share structural and functional similarities with the inner ear hair cells, making it an excellent model to studying hair cell development and function (Sheets et al., 2021). Additionally, morpholino oligonucleotides (MOs), a rapid and efficient gene-editing tool, have been widely used to validate candidate deafness genes (Gao et al., 2018; Li et al., 2023). However, the role of *mfsd3* in zebrafish auditory function and embryonic development remains unexplored.

In this study, we examined the expression levels of *mfsd3* throughout various embryonic developmental stages in zebrafish. We further demonstrated that MO-mediated knockdown of *mfsd3* in zebrafish led to auditory organ dysfunction, developmental defects and potential disruption of the Wnt/β-catenin signaling pathway.

## 2 Materials and methods

### 2.1 RNA extraction and quantitative real-time PCR (qRT-PCR)

Total RNA was extracted from 4–10 embryos per group using Trizol (Roche) according to the manufacturer’s protocols. cDNA was synthesized using the PrimeScript RT reagent Kit with gDNA Eraser (Takara). qPCR was performed in triplicates on a Realplex system (Eppendorf) using iQ SYBR Green Supermix (Bio-rad), with *ef1α* as the endogenous control. Relative expression levels were calculated using the 2^−ΔΔCT^ method. Primer sequences are listed in Supplementary Table S2.

### 2.2 Zebrafish husbandry and *mfsd3* MO knockdown

Wild-type AB strain and Tg (Brn3c:mGFP) S356T transgenic zebrafish (provided by Prof. Hua-Wei Li, Fudan University) were maintained at 28.5 °C under a 14 h/10 h light/dark cycle. For the latter, membrane-targeted GFP is specifically expressed in hair cells under control of the brn3c promoter. Antisense MO (Gene Tools, LLC, United States of America) was microinjected into fertilized embryos at one-cell-stage following standard protocols (Nasevicius and Ekker, 2000). Two specific morpholino antisense strategies were employed to target zebrafish *mfsd3* RNA, preventing either translation of the gene (ATG-MO) or proper splicing of exon2 (E2I2-MO). The sequences for *mfsd3-*ATG-MO and *mfsd3-*E2I2-MO were 5′-GAA​CAC​CAG​CTT​GTC​GTT​CAT​CAT​G-3′ and 5′-ACA​ACA​AAA​CAC​ACT​CCC​TAC​CTG​T-3′, respectively. The sequence for the standard control morpholino was 5′-CCT​CTT​ACC​TCA​GTT​ACA​ATT​TAT​A-3’ (Gene Tools). The optimal dose (4ng/embryo) of MOs was determined through dose-response experiments. Knockdown efficacy was verified by reverse transcription PCR (RT-PCR) using primers spanning *mfsd3* exons 1–4 (forward: 5′-GTG​TGG​AGG​AAG​CTG​TTA​TC-3’; reverser: 5′-AGC​ACC​GCC​ATA​ACT​TTC-3′).

### 2.3 Inner ear morphology and hair cells quantification

At 3 dpf, Tg (Brn3c:mGFP) zebrafish embryos were anesthetized using 0.016% MS-222 (tricaine methanesulfonate; Sigma-Aldrich, St. Louis, MO, United States of America). The anesthetized larvae were then laterally positioned (anterior to the left, posterior to the right, and dorsal side up) and immobilized in 3% methylcellulose within a depression slide for fluorescence microscopic observation. The larvae were divided into three groups: Control-MO, *mfsd3-*E2I2-MO and *mfsd3-*ATG-MO. The inner ear morphology and hair cells in lateral line neuromasts were quantified analyzed: The number of hair cells in lateral line neuromasts, otic vesicle area (×10^3^ μm^2^), otolith area (μm^2^) and diameter (μm). The maximum diameter or cross-sectional area (the maximum projection) of both otoliths from each zebrafish was measured, using NIS-Elements D4.6 measurement software integrated into the Nikon SMZ18. The average value was then calculated to represent the otolith diameter or area for each individual. Ten zebrafish per group were randomly selected for measurement, followed by quantitative statistical analysis.

### 2.4 Behavioral analysis

5-dpf larvae (n = 10/group) were acclimated for 15 min in 96-well plates (0.2 mL/well) and tracked for 30 min using Ethovision XT software (Noldus) (Zhao et al., 2012). Movement parameters analyzed included: Total distance traveled, average velocity, mobility percentage, maximum acceleration. A 0.2 mm movement threshold was applied to filter system noise. The larvae were divided into three groups: Control-MO, *mfsd3-*E2I2-MO and *mfsd3-*ATG-MO. Ten zebrafish per group were randomly selected for monitoring, followed by quantitative statistical analysis.

### 2.5 Image acquisition and statistical analysis

Images were acquired using a Nikon SMZ18 fluorescence microscope and processed with Adobe Photoshop 7.0 software (Adobe, San Jose, California) for optimize visualization. Quantitative analyses were performed using NIS-Elements D4.6 (Japan) and ImageJ (U.S. National Institutes of Health, Bethesda, MD, United States of America). Inverted fluorescent images were utilized for processing. Data were presented as mean ± SEM. Ten animals from each treatment group underwent quantification with averaging performed on total signal per animal. And GraphPad Prism 5.0 (GraphPad Software, San Diego, CA) was used for statistical analysis and graphical representation of the data. Statistical analyses of the comparison between two groups (control MO and E2I2-MO) were performed by Student’s t-test. One-way analysis of variance (ANOVA) was used for comparison among three or more groups. For further pairwise comparisons among groups, the Bonferroni’s multiple comparison test (among control MO, ATG-MO and E2I2-MO) and Tukey’s multiple comparisons test (*mfsd3* expression level among different developmental stages) were utilized. Statistical significance is indicated as *P < 0.05, **P < 0.01, ***P < 0.001 and ****P < 0.0001.

## 3 Results

### 3.1 Knockdown of *mfsd3* causes inner ear defects in zebrafish

**FIGURE 1:**
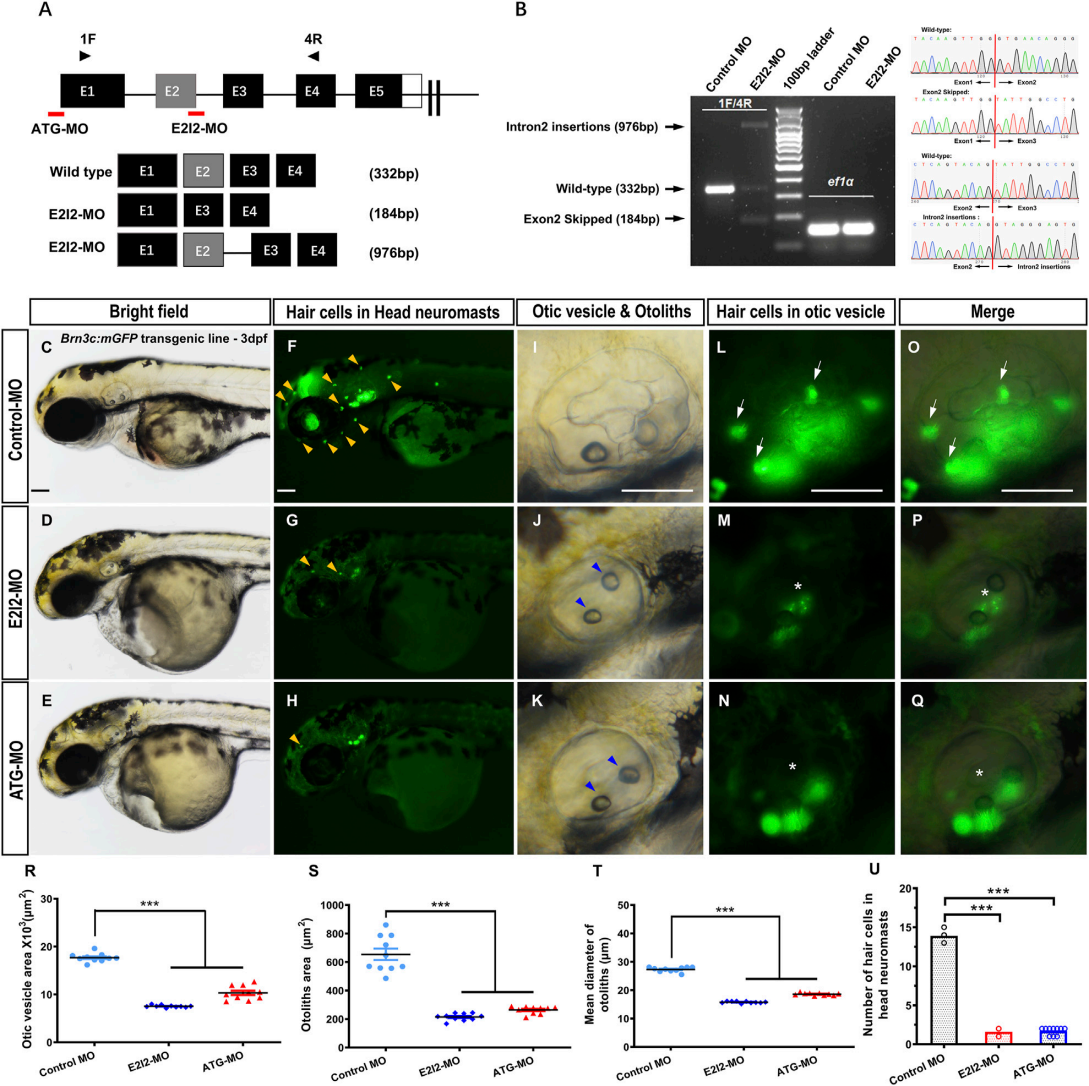
Phenotypes of auditory system in *mfsd3* zebrafish morphants. **(A)** Schematic of two MO targeting strategies: ATG-MO blocks translation initiation while E2I2-MO disrupts exon 2-intron 2 splicing. Primers pairs (1F/4R) detect wild-type transcripts or aberrant splicing products containing retained intron 2 or skipped exon 2. **(B)** Left: RT-PCR analysis of *mfsd3* transcript from 1 dpf embryos injected with control-MO or E2I2-MO (4 ng). Aberrant splicing product (intron 2 retention and exon2 skipping) are indicated by shifted band patterns. Right: Sanger sequencing confirmation of wild-type sequence, intron 2-inserted transcript and exon2-skipped transcript. **(C**–**Q)** Gross morphology of head neuromasts and inner ear in Tg (Brn3c: mGFP) embryos at 3-dpf. Compared to control MO, Mfsd3 deficiency led to reduced hair cell numbers in head neuromasts (**(G,H),** yellow arrowheads), hypoplastic otic vesicle **(J,K)**, malformed otoliths (**(J,K)**, blue arrowheads) and damaged otic vesicle hair cells (**(M–N)**, **(P–Q)**, asterisk). **(R–T)** Quantitative measurements demonstrated significant reduction in: otic vesicle area **(R)**, otoliths area **(S)** and mean otolith diameter **(T)**. ***P < 0.001 (n = 10; ANOVA). **(U)** Quantitative of hair cell numbers in head neuromasts showing significant decrease in morphants. Scale bar, 100 μm; Scatter plot with bars; ***P < 0.001 (n = 10; ANOVA). dpf, days post fertilization.

We first confirmed *mfsd3* in zebrafish via qRT-PCR at multiple time points prior to 6 dpf. Additionally, we obtained the relative expression level (TPM) of *mfsd3* across 18 developmental stages from the Expression Atlas database (http://www.ebi.ac.uk/gxa/experiments/E-ERAD-475), which were derived from poly(A)-selected RNA-seq data (White et al., 2017). Both datasets revealed a significant decline in *mfsd3* mRNA levels after 6 hpf, suggesting its potential involvement in early zebrafish development (Supplementary Figure S2). The efficiency of *mfsd3* knockdown was verified by RT-PCR (Figures 1A,B).

**FIGURE 2:**
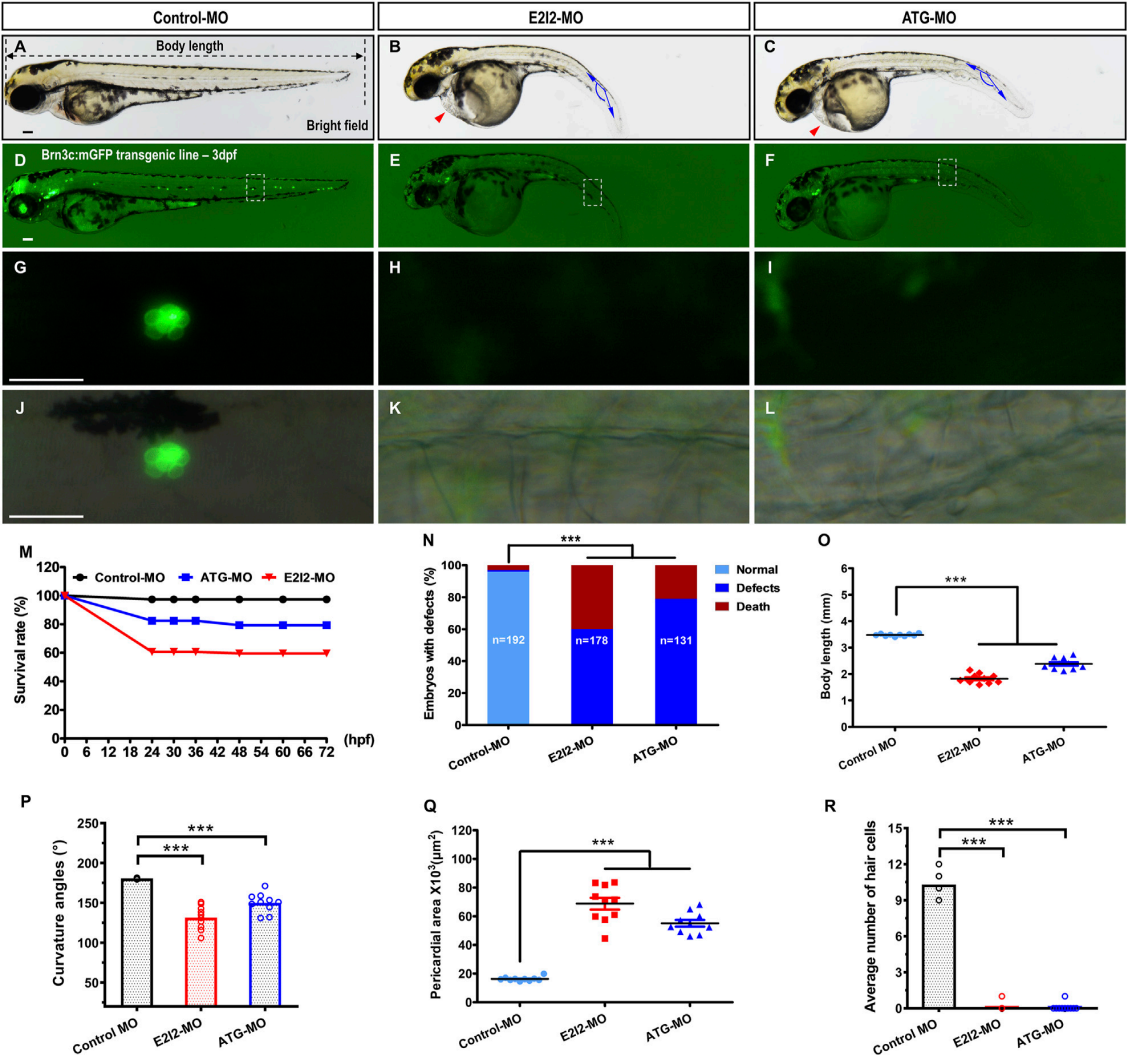
Morphological defects and hair cells loss in Mfsd3-deficient zebrafish. **(A–C)** Compared with control MO, the *mfsd3* knockdown MO showing significant body shortening **(B,C)**, axis curvature **(B,C)** and pericardial edema (**(B,C)**, red arrowheads). **(D**–**L)** Live imaging of Brn3c: mGFP transgenic embryo at 3-dpf showed GFP expression from RGCs (retinal ganglion cells) and neuromasts (green dots) of the posterior lateral line and head. Zebrafish under control exhibited normal hair cells number **(D)**. Conversely, significantly decreased hair cells of the posterior lateral line were observed in *mfsd3* morphants **(E,F)**. The white boxed regions are presented in amplified form in the lower panels **(G**–**L)** GFP fluorescence signal alone **(G**–**I)** and GFP fluorescence signal merged with bright field image **(J**–**L)**. Morphometric analyses were utilized to quantify the fluorescence particle signal of hair cells. **(M)** A time-course plot of survival rates in control vs. *mfsd3* morphants for 3 days. **(N)** The percentage of embryos with development defects in control vs. *mfsd3* morphants. **(O,P)** Quantitative analysis of body length **(O)** and curvature angle **(P)** of embryos. (n = 10; ANOVA; ***P < 0.001). **(Q)** Quantitative analysis of the pericardial area of embryos. **(R)** Quantitative analysis of the average number of hair cells at posterior lateral line. Scale bar, 100 μm; Scatter plot with bars (n = 10; ANOVA; **P < 0.0001). dpf, days post fertilization.

Compared to wild-type (WT) controls, *mfsd3* morphants exhibited a reduced number of hair cells in both head neuromasts and posterior lateral line neuromasts (Figures 1F–H,L–Q,U; Figures 2D–L,R). At 3 dpf, the average hair cell counts in head neuromasts were: 1.6 ± 0.52 (E2I2-MO), 1.7 ± 0.48 (ATG-MO) and 13.9 ± 0.57 (WT); the average hair cell counts in posterior lateral line neuromasts were: 0.1 ± 0.32 (E2I2-MO and ATG-MO) and 10.2 ± 0.92 (WT). Smaller otic vesicles and otoliths were observed in the morphant than in the WT (Figures 1C–E, I–K, R–T). The average areas (×10^3^, μm^2^) of otic vesicles were 7.5 ± 0.25 (E2I2-MO), 10.3 ± 1.45 (ATG-MO) and 17.7 ± 0.89 (WT). The average areas (μm^2^) of otoliths were 214.4 ± 24.79 (E2I2-MO), 263.3 ± 25.90 (ATG-MO) and 654.0 ± 126.50 (WT). The average diameters (μm) of otoliths were 15.8 ± 0.32 (E2I2-MO), 18.6 ± 0.45 (ATG-MO) and 27.3 ± 0.82 (WT). These data indicate that Mfsd3 deficiency leads to otic vesicle and otolith hypoplasia, as well as hair cells loss in lateral line system.

### 3.2 *mfsd3* knockdown results in decreased survival, morphological defects and pericardial edema

At 3 dpf, both *mfsd3*-E2I2 and *mfsd3*-ATG morphants exhibited nearly identical phenotypes, including morphological abnormalities and pericardial edema (Figure 2; Supplementary Figure S3), along with significantly lower survival rates: 59.55% (E2I2-MO, n = 178 embryos), 79.39% (ATG-MO, n = 131 embryos) and 97.40% (WT controls, n = 192 embryos) (Figure 2M). Loss of Mfsd3 also caused pronounced developmental defects (Figure 2N), including shortened body length, curved body axis and pericardial edema (Figures 2A–C, O–Q). The mean body lengths (mm) were 1.826 (E2I2-MO), 2.382 (ATG-MO) and 3.477 (WT, n = 10). The mean body axis curvatures (°) were 131.5 (E2I2-MO), 149.9 (ATG-MO) and 180.6 (WT, n = 10). The mean pericardial areas (×10^3^, μm^2^) were 68.81 (E2I2-MO), 55.16 (ATG-MO) and 16.29 (WT, n = 10). These findings indicate that the *mfsd3* gene plays a critical role in early zebrafish development.

### 3.3 *mfsd3* knockdown results in locomotor defects

**FIGURE 3:**
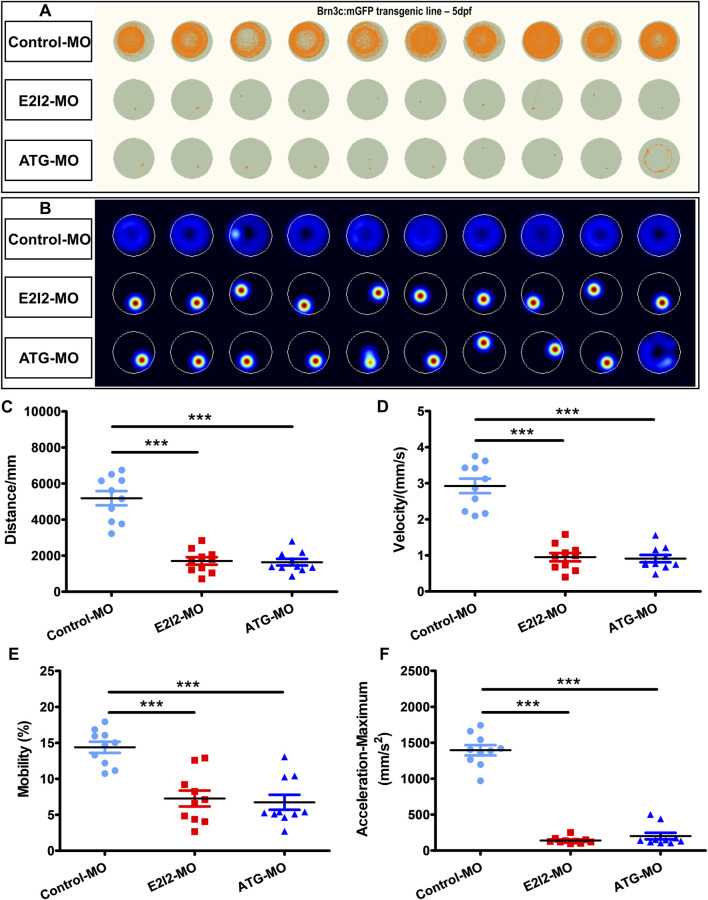
Locomotor capacity was reduced in *mfsd3* zebrafish morphants. **(A,B)** The digital tracks and heatmap image in larvae from control-MO and *mfsd3*-MO injected groups at 5-dpf. Each behavioral test consisted of ten larvae each placed into individual wells. **(C–F)** The statistical analyses showed significantly reduced total movement distance **(C)**, velocity **(D)**, mobility **(E)** and maximum acceleration **(F)** in *mfsd3* morphants compared to control-MO injected groups (n = 10; ANOVA; ***P < 0.001). dpf, days post fertilization.

Given the morphological abnormalities observed in *mfsd3* morphants at 3 dpf, we further assessed their swimming behavior at 5 dpf. Both E2I2-MO and ATG-MO morphants exhibited significant reduction in total swimming distance, velocity, mobility and maximum acceleration compared to WT (Figure 3, n = 10). Mean distances (mm) were 1708 (E2I2-MO), 1,639 (ATG-MO) and 5,185 (WT). Mean velocities (μm/s) were 949.5 (E2I2-MO), 911.2 (ATG-MO) and 2,925 (WT). Mean mobilities (%) were 7.25 (E2I2-MO), 6.73 (ATG-MO) and 14.38 (WT). The average maximum accelerations (mm/s^2^) were 139.9 (E2I2-MO), 201.7 (ATG-MO) and 1,396 (WT). These deficits likely stem from tail dyskinesia, which may be secondary to the abnormal body axis morphology.

### 3.4 *mfsd3* knockdown potentially disrupts the wnt/β-catenin signaling pathway

In this study, targeted knockdown of *mfsd3* in zebrafish embryos resulted in multiple developmental defects, including pericardial edema, body axis curvature, and hair cells loss. These phenotypes suggest potential dysregulation of the Wnt/β-catenin pathway (Bellipanni et al., 2006; Ueno et al., 2007; Ma and Raible, 2009). Since both morphant groups exhibited similar defects, we focused on E2I2-MO for pathway analysis.

**FIGURE 4:**
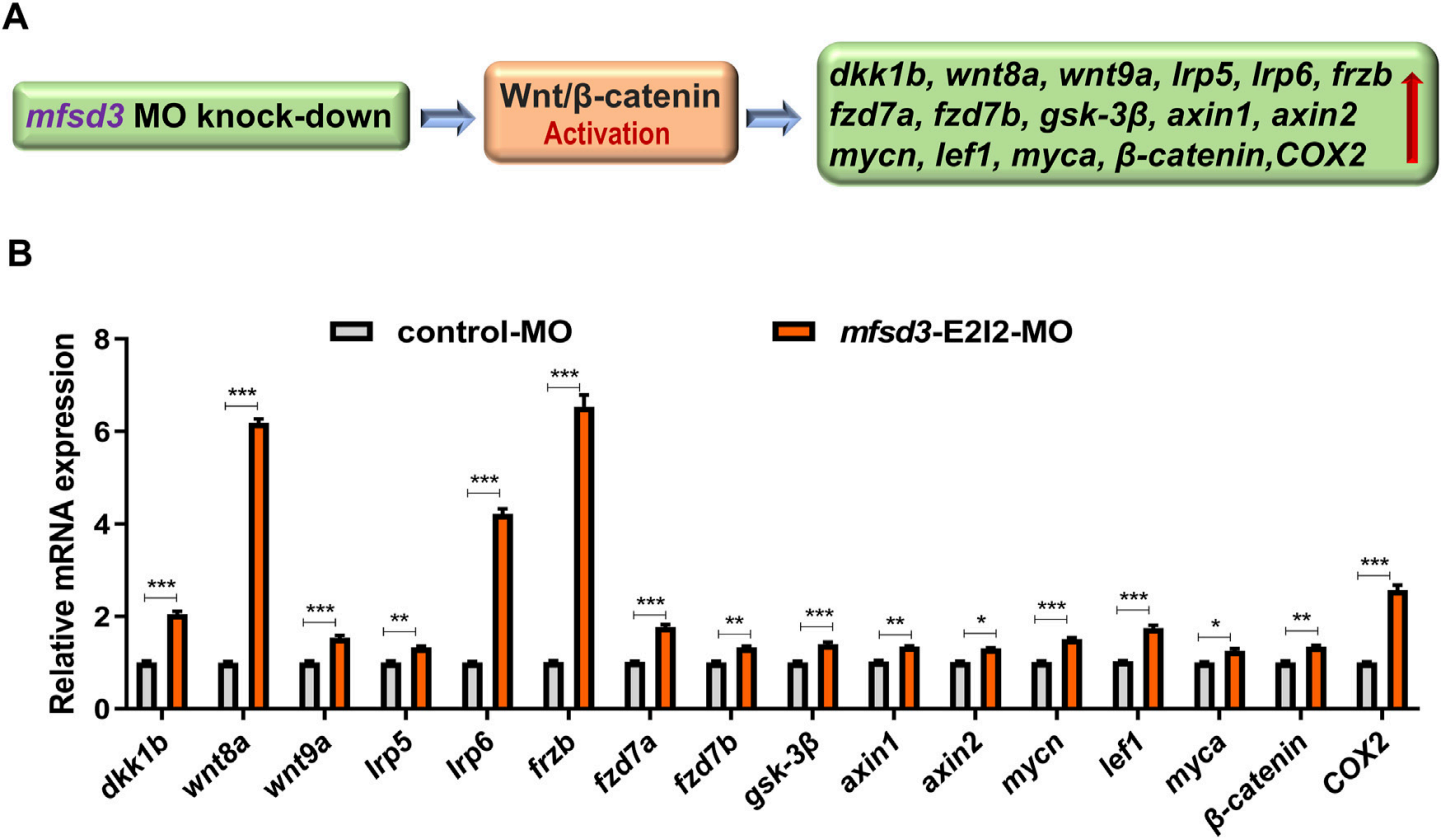
Zebrafish with Mfsd3 deficiency exhibited upregulated Wnt/β-catenin signaling activity. **(A)** Schematic diagram depicting the potential regulatory action of *mfsd3* on the Wnt/β-catenin signaling pathway. **(B)** Wnt/β-catenin signaling pathway was upregulated in *mfsd3* morphants. Endogenous *dkk1b*, *wnt8a*, *wnt9a*, *lrp5*, *lrp6*, *frzb*, *fzd7a*, *fzd7b*, *gsk-3β*, *axin1*, *axin2*, *mycn*, *lef1*, *myca*, *β-catenin* and *COX2* in control and *mfsd3* morphants measured by qRT-PCR assay (n = 6–10 individual embryos). The *dkk1b*, *wnt8a, lrp6, frzb* and *COX2* were all upregulated significantly in the *mfsd3* morphants group. ***P < 0.001; **P < 0.01; *P < 0.05.

qRT-PCR revealed upregulation of key Wnt/β-catenin components in *mfsd3* morphants, including *dkk1b, wnt8a, wnt9a, lrp5, lrp6, frzb, fzd7a, fzd7b, gsk-3β, axin1, axin2, mycn, lef1, myca, β-catenin and COX2*. Notably, *dkk1b, wnt8a, lrp6, frzb and COX2* showed the most pronounced changes (Figure 4)*.* These results suggest that Mfsd3 deficiency may disrupt Wnt/β-catenin signaling through direct or indirect mechanisms.

## 4 Discussion

MFSD3 belongs to the acetyl-CoA transporter family SLC33 and shares 19.5% sequence homology with SLC33A1 (Perland et al., 2017b). The National Center for Biotechnology Information (NCBI) database annotations predict MFSD3 to function as a solute: proton symporter involved in proton transmembrane transport while serving as an integral membrane component. The Kyoto Encyclopaedia of Genes and Genomes (KEGG) database further supports its potential role as an acetyl-CoA transporter. Expression data from Mouse Genome Informatics (MGI) and Shared Harvard Inner Ear Laboratory (SHIELD) databases revealed *Mfsd3* expression in mouse cochlea structures, including the organ of Corti, otic progenitor cells, spiral ganglion and vestibular ganglion. Additionally, we analyzed single-cell RNA-sequencing data from three independent published studies via Seurat in R 4.3.3 (Petitpré et al., 2018; Shrestha et al., 2018; Petitpré et al., 2022). This analysis further demonstrated an age-dependent increase in *Mfsd3* expression in the spiral ganglion neurons of mice cochlea (Supplementary Figure S4).

Current research on *MFSD3* remains limited, with only five international studies published to date (Perland et al., 2017b; Li et al., 2019; Nicoletti et al., 2020; Xu et al., 2021; Shigemizu et al., 2022). Among these, three suggest potential disease associations through clinical data screening, though the protein’s exact function and physiological mechanism remain undefined. Key findings demonstrated that *Mfsd3* shows abundant expression in the nervous system, liver, kidneys and ovaries of WT mice (Perland et al., 2017b), with potential links to neurodegenerative disorders and tumors (Li et al., 2019; Shigemizu et al., 2022). Notably, MFSD3 localized specifically to neuronal plasma membranes in mice brains (Perland et al., 2017b), with *Mfsd3* mRNA levels decreasing throughout the brain under high-fat diets yet showing brainstem-specific upregulation during starvation (Perland et al., 2017b). Transkingdom Network analysis connects *Mfsd3* to liver fatty acid metabolism in mouse (Rodrigues et al., 2021), while clinical evidence associates *MFSD3* hypermethylation with post-bariatric weight regain (Nicoletti et al., 2020). These collective findings position *MFSD3* as a potential regulator of nutrition intake, lipid metabolism and energetic homeostasis. Given the exceptional sensitivity of the cochlea to local lipid metabolism, disturbances in this metabolic process can induce hearing impairment via the degeneration or apoptosis of hair cells (Saito et al., 1986; Wu et al., 2012; Yao et al., 2019). This underpins the potential involvement of MFSD3 in auditory function.

Our investigation of zebrafish *mfsd3* revealed a distinct temporal expression pattern, with peak expression occurring between 0.75 and 6 hpf. Given that zygotic genome activation (ZGA) initiates at 2.75-3hpf (Shen et al., 2022), we interpret transcripts before 3hpf as maternal in origin, with zygotic transcription commencing thereafter. The subsequent sharp decline after 6 hpf followed by a sustained low-level expression implies coordinated contributions from both maternal and zygotic *mfsd3* during early development. However, the mechanism driving the pronounced expression surge during the early zygotic stage (0–0.75 hpf) requires further elucidation.

Functional characterization using two distinct MOs (ATG blocker and E2I2 splice blocker) yielded consistent phenotypes in zebrafish, including otic vesicle and otoliths hypoplasia, hair cells loss in lateral line, and motor deficit. The observed hair cells reduction might be attributed to the complete absence of entire neuromasts, a hypothesis that necessitates validation through future immunostaining with cell-type specific markers like SOX2 (supporting cells) and BrdU (proliferated cells). These auditory developmental defects suggest *MFSD3*’s potential role in auditory function, while acknowledging possible interspecies differences between zebrafish and mammalian systems. Concurrent phenotypes of reduced embryo viability, morphological abnormalities and pericardial edema underscore the *mfsd3*’s importance in early development of zebrafish. Notably, our use of low-dose MOs (4ng/injection) minimizes potential off-target effects, as background phenotypes typically require higher doses (≥40 ng) (Söllner et al., 2003). This represents the first reported zebrafish model of Mfsd3 deficiency. Building on its predicted acetyl-CoA transporter activity (Perland et al., 2017b), we propose that Mfsd3 loss may impair acetyl-CoA membrane transport, potentially disrupting energy production and lipid metabolism while promoting pathological lipid accumulation in auditory structures, which was a hypothesis requiring further experimental validation.

The evolutionarily conserved Wnt/β-catenin pathway has been confirmed to regulate both development and oncogenesis (Moon et al., 2002). Studies have shown that the alterations to Wnt/β-catenin signaling induced body axis and cardiac defects in zebrafish (Bellipanni et al., 2006; Wan et al., 2021), which was also observed in our study. Growing evidence highlights this pathway’s critical role in auditory system development (Chai et al., 2012; Munnamalai and Fekete, 2013), particularly in cochlea sensory epithelium differentiation and neuromast progenitor proliferation (Shi et al., 2014; Tang et al., 2019). While our work implicates potential *mfsd3* in Wnt/β-catenin signaling regulation, definitive confirmation awaits further rescue-based functional validation.

Several limitations and future directions merit consideration. First, potential MO-induced mosaicism necessitates CRISPR/Cas9-generated knockout models for phenotype confirmation. Second, phenotypic analysis requires refinement through: 1) left-right patterning assessment via of Kupffer’s vesicle and cardiac looping analysis (Essner et al., 2005; Bowley et al., 2022); 2) lateral line primordium development assessment and migration studies using immunohistochemistry; and 3) comprehensive evaluation of motor dysfunction through neuromuscular assessment. Third, given the extensive crosstalk among Wnt, FGF, Notch, BMP and SHH pathways during inner ear development (Munnamalai and Fekete, 2013; Roccio and Edge, 2019), future RNA-seq analysis should explore *mfsd3*’s broader signaling impacts beyond Wnt/β-catenin. In addition, to characterize the expression pattern of *Mfsd3* in cochlea tissue, we initially tested a commercially available MFSD3 antibody (AV51707, Sigma-Aldrich, species reactivity: human and mouse) in HEK293T cells transfected with the pcDNA3.1-CMV-*MFSD3* (human-NM_138431.3)-EGFP plasmid. However, the antibody exhibited poor specificity (Supplementary Figure S5). Future studies are essential to examine the *Mfsd3* spatiotemporal expression pattern in the mice inner ears through RNA *in situ* hybridization, as well as to identify whether the inner ear structures and hearing function are impaired in murine models with aberrant Mfsd3.

## 5 Conclusion

Our study implicates *MFSD3* in auditory function and zebrafish embryogenesis. We identified predominant early embryonic expression of *mfsd3* gene in zebrafish. Functional knockdown of *mfsd3* in zebrafish produced diverse abnormalities, affecting auditory structures, embryonic morphology, cardiac development and motor behavior, along with potential Wnt/β-catenin pathway upregulation. These findings establish foundational insights while highlighting the need for deeper mechanistic exploration of *MFSD3*’s role in auditory and developmental biology.

## Data Availability

The original contributions presented in the study are included in the article/Supplementary Material, further inquiries can be directed to the corresponding authors.

## Abbreviations

MFSD3, Major facilitator superfamily domain containing protein 3; SLC, solute carrier; SLC33A2, solute carrier family 33 member 2; MFS, major facilitator superfamily; HL, hearing loss; SNHL, sensorineural hearing loss; ACMG, American College of Medical Genetics and Genomics; AMP, Association for Molecular Pathology; VUS, variant of uncertain significance; SEM, standard error of the mean; NCBI, National Center for Biotechnology Information; KEGG, Kyoto Encyclopaedia of Genes and Genomes; MGI, Mouse Genome Informatics; SHIELD, Shared Harvard Inner Ear Laboratory Database; ZGA, zygotic genome activation; KV, Kupffer’s vesicle; FGF, fibroblast growth factor; BMP, bone morphogenetic protein; SHH, sonic hedgehog.
