# Supplementary figures and images for: Functional characterization of MFSD3 in auditory system and zebrafish embryogenesis
Source: Front Genet. 2025 Sep 15;16:1634493. doi: 10.3389/fgene.2025.1634493 (PMC12477447; doi:10.3389/fgene.2025.1634493)

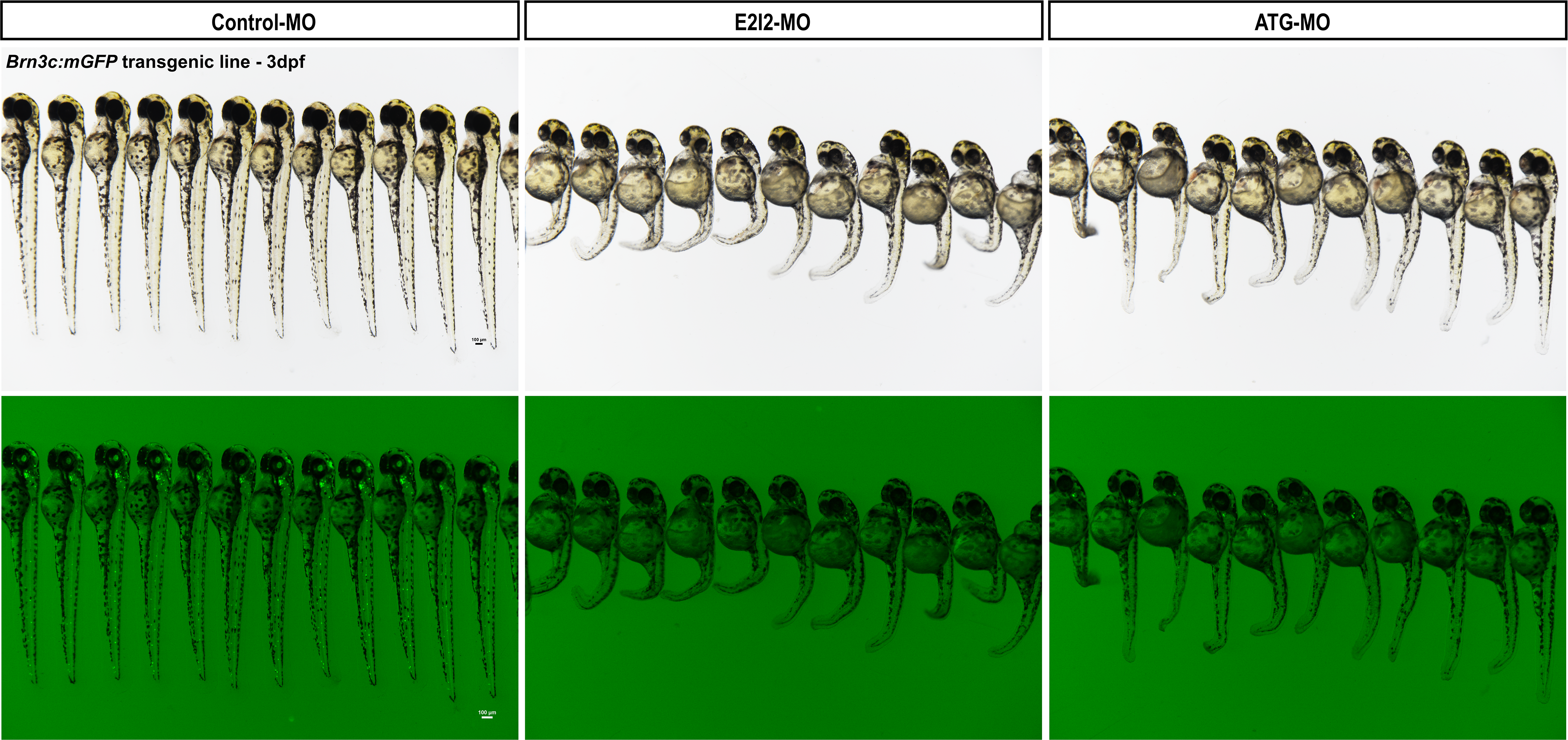

Supplement: Supplementary file 1 [file Image3.tif]

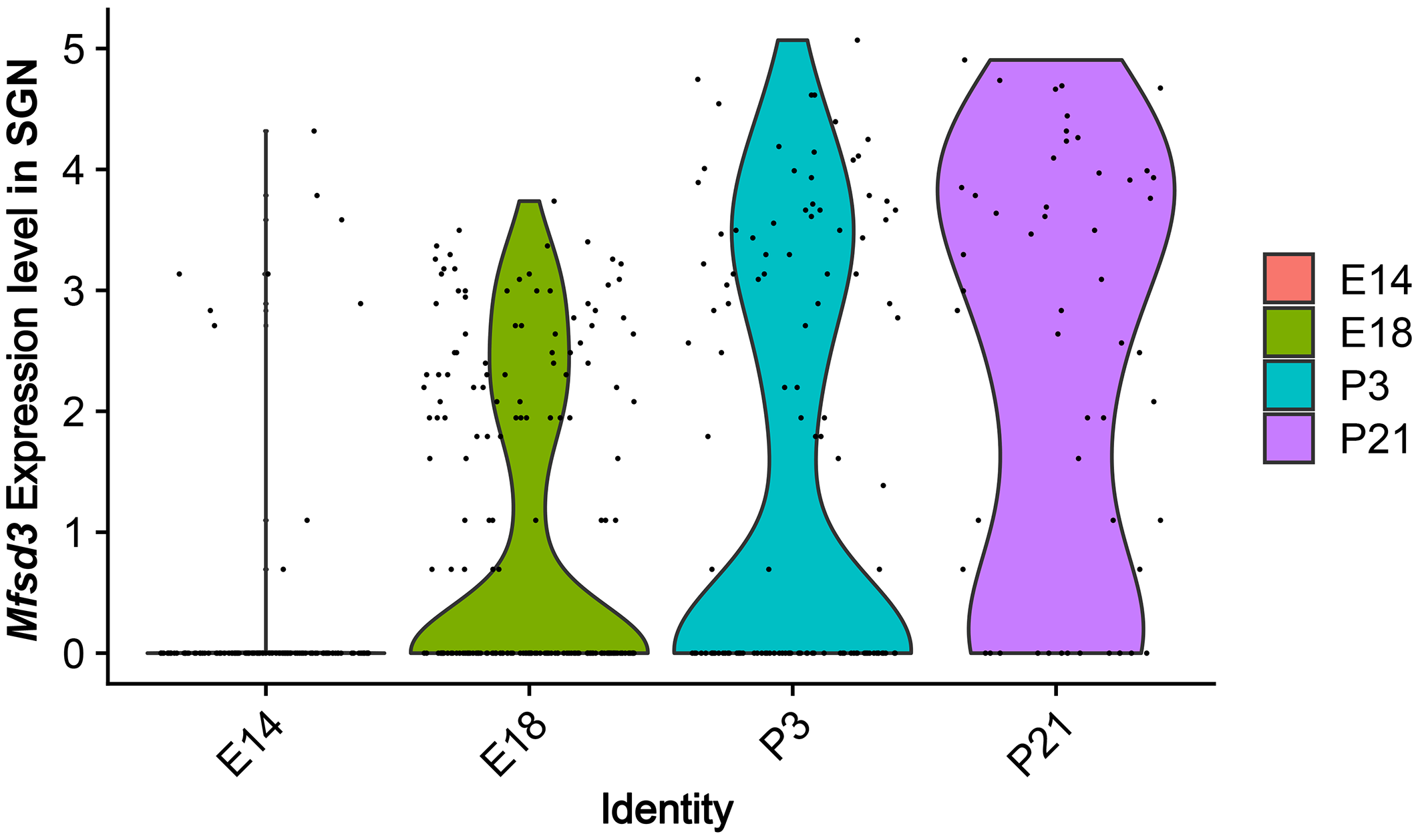

Supplement: Supplementary file 2 [file Image4.tif]

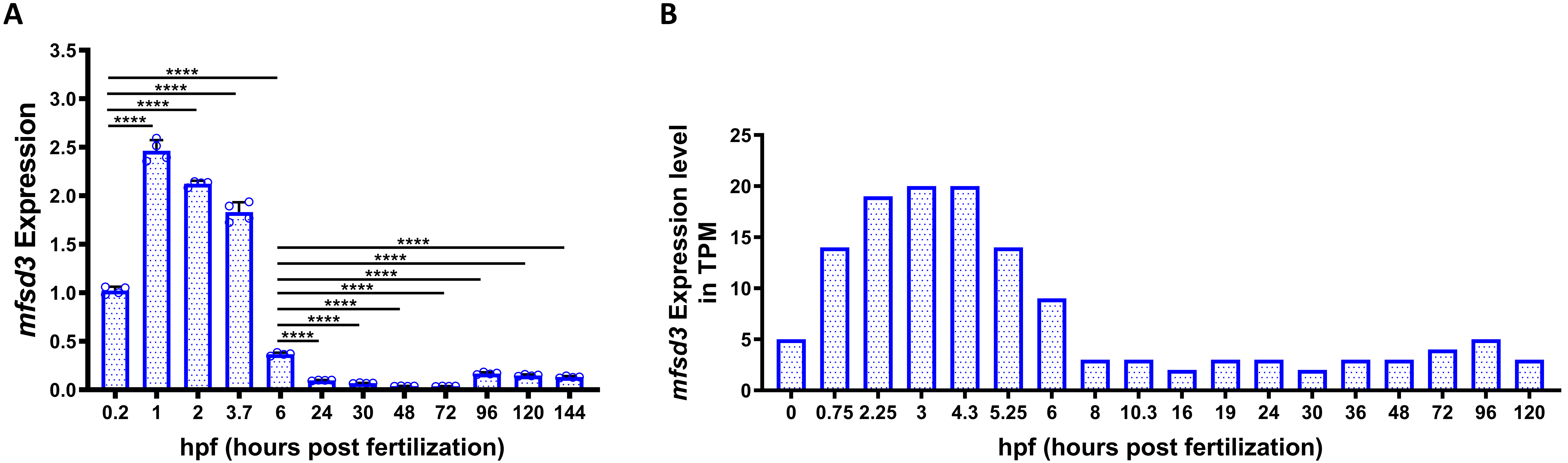

Supplement: Supplementary file 3 [file Image2.tif]

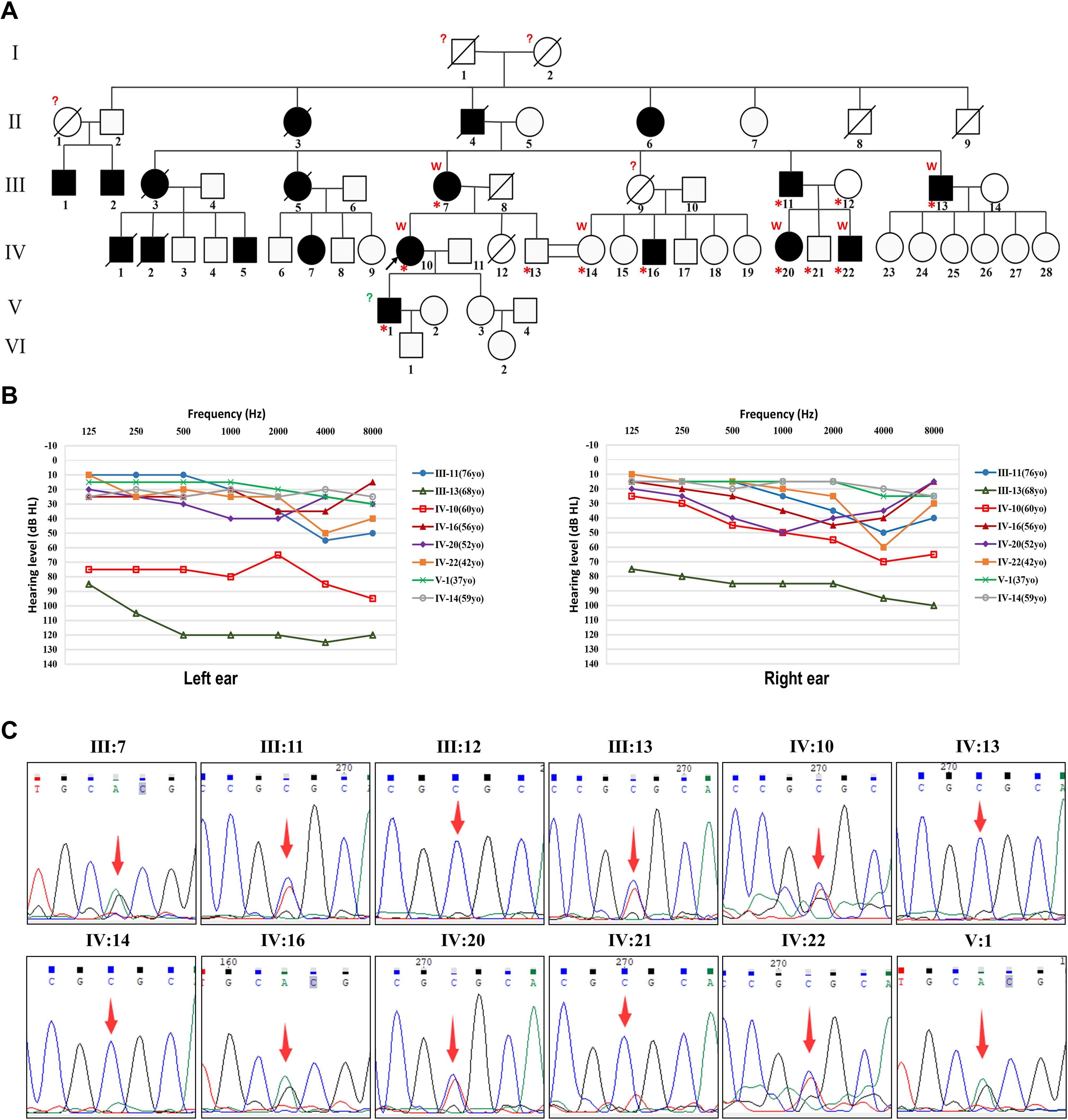

Supplement: Supplementary file 4 [file Image1.tif]

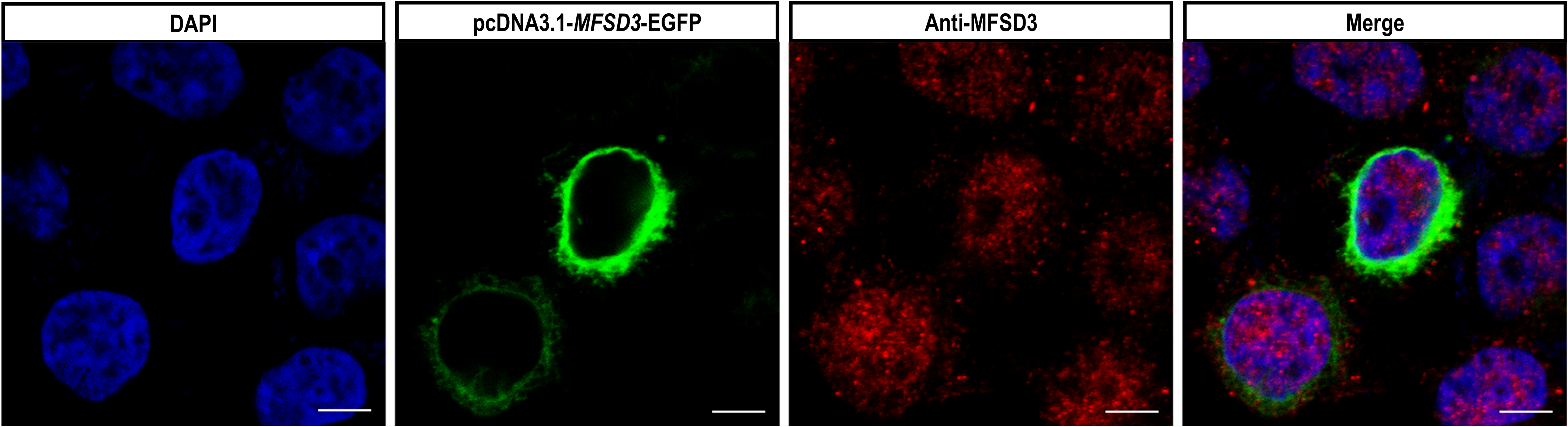

Supplement: Supplementary file 6 [file Image5.tif]
