# Supplementary material for: Functional characterization of MFSD3 in auditory system and zebrafish embryogenesis
Source: Front Genet. 2025 Sep 15;16:1634493. doi: 10.3389/fgene.2025.1634493 (PMC12477447; doi:10.3389/fgene.2025.1634493)
Supplement: Supplementary file 5 [file Supplementaryfile1.doc]

Supplementary Material

# A Chinese Han ADNSHL Family with a *MFSD3* Missense Variant

## Clinical characterization of the affected individuals

We investigated a six-generation Chinese family (No. HBZ02; Supplementary Figure 1A) with segregating autosomal-dominant, non-syndromic, bilateral, symmetric progressive sensorineural hearing loss (ADNSHL) that initiated in the fifth decade of life and was often accompanied by tinnitus. The affected individuals reported a gradual progression of hearing loss, initially affecting high frequencies and subsequently extending to all frequencies (Supplementary Figure 1B). The proband (Ⅳ-10) and other two family members (Ⅲ-13, Ⅳ-16) also experienced vertigo (Supplementary Table 2). There were no indications of a correlation between hearing loss and infections, noise exposure or ototoxic drugs. Results from ABR and DPOAE tests ruled out auditory neuropathy spectrum disorders. The affected individuals did not exhibit any significant delays in intellectual and motor development, nor did they show any signs of other organs abnormalities or morphological features indicative of syndromic forms of hearing loss. High-resolution CT scans of the temporal bone in affected individuals revealed normal findings, excluding malformations in the middle and inner ear.

## Identification of a novel heterozygous missense variant in *MFSD3*

DNA samples were collected from six unaffected and eight affected individuals of family HBZ02. The proband (Ⅳ-10) was pre-screened and found to be negative for variants in *GJB2*, *SLC26A4* and mtDNA12SrRNA via direct sequencing. Considering the history of vertigo in some members (Ⅲ-13, Ⅳ-10) of this family, the whole-exome sequencing of the *COCH* gene was also performed, but no positive variant was found. To identify the genetic cause of hearing loss in the family, we subsequently conducted whole-genome sequencing on five affected (Ⅲ-7, Ⅲ-13, Ⅳ-10, Ⅳ-20, Ⅳ-22) and one unaffected (Ⅳ-13) subject. Through combining with PCR-Sanger sequencing, a missense variant *MFSD3* c.608C>T (p. Ala203Val) was finally identified, which cosegregated in the genotype and phenotype in this pedigree (Supplementary Figure 1C).

# Supplementary Tables

**Supplementary Table 1. Phenotypes of the affected individuals in family HBZ02.**

| **Patient ID** | **Age**  **(yrs)** | **Gender** | **Age at onset**  **(yrs)** | **Audiometric threshold curve** | **Progressive** | **Laterality** | **Tinnitus** | **Vertigo** |
| --- | --- | --- | --- | --- | --- | --- | --- | --- |
| III:7 | 83 | Female | 52 | All-frequency HL | Yes | Bilateral | Yes | No |
| III:11 | 76 | Male | — | High-frequency HL | Yes | Bilateral | Yes | No |
| III:13 | 68 | Male | 53 | All-frequency HL | Yes | Bilateral | Yes | Yes |
| IV:10 | 60 | Female | 50 | All-frequency HL | Yes | Bilateral | Yes | Yes |
| IV:16 | 56 | Male | 52 | Mid-High-frequency HL | Yes | Bilateral | Yes | Yes |
| IV:20 | 52 | Female | 50 | Mid-High-frequency HL | Yes**^*^** | Bilateral | Yes | No |
| IV:22 | 42 | Male | — | High-frequency HL | Yes**^*^** | Bilateral | Yes **^#^** | No |
| V:1 | 37 | Male | — | High-frequency HL | — | Unilateral | No | No |

HL, hearing loss; —, unknown; yrs, years of age; *, follow-up: self-reported progression of HL; #, follow-up: onset of tinnitus at 51yrs.

**Supplementary Table 2. qRT-PCR primers for zebrafish**

| **Gene** | **Forward Primer 5'-3'** | **Reverse Primer 5'-3'** |
| --- | --- | --- |
| *mfsd3* | TTGTTGCCATGGCCTTT | TCTGGAAGACCATGCTTATTG |
| *dkk1b* | TCGCCCATGAAAACTCTACTG | TGGACCAAAAGTGACGAGC |
| *wnt8a* | CTCTACTCACAAAGGCTTGAGAA | CAAGACTGCAGTTTCTGGTTAAAG |
| *wnt9a* | CGCTACAAAATGCTGGATGG | TCAGTGGCAGAATGGACAG |
| *lrp5* | GCTCCTCTCTATGACCGAAAC | ACGGGCTGTTGGATGAATAA |
| *lrp6* | TGAGGTGCTGTTCTTCAGTAATC | GTTGGAGTCCTCGATCACAATC |
| *frzb* | TCCGGCTGTCTGTGTCCTCCGCTCAC | GTCCTCCTCCCGTTTGCAGCCTGGTC |
| *fzd7a* | CCCGCCACTATCGTGATCGCCTGCTA | CCGCGTAACAGTCCGCACGAGACATTC |
| *fzd7b* | CGAGCGCAAAAGGAGGCAGAT | CTGGCGCGTACATGGAGCAGA |
| *β-catenin* | CTGCACATTCTAGCCAGAGAC | CCTTATCCTGAGCCAGTTCAC |
| *gsk-3β* | ATCTTAATCCCCGCTCATGC | CAGGTTGAGGTGTTAGAGGC |
| *axin1* | GACATGGAGAGGAACCAGAAG | ATGACCCTGAGCTTTCTTGG |
| *axin2* | CTTACCCTCGGACACTTCAAG | CCCTCATACATTGGCAGAACTG |
| *lef1* | AATTTCTATCCCCTTTCCCCG | TGCTCCTGTTTCACCTGTG |
| *mycn* | CAATTGCCTCAAGTCAGTGC | ACGACATCAATCTCTTCCTCTTC |
| *myca* | GACACTCCACCTAACAGCTC | TCGCTTTTCCACAGTCACC |
| *COX2* | GTACCAACCCAAGACCTCAC | TGATGGAACAGCTCAAGAGTG |
| *ef1α* | GGAAATTCGAGACCAGCAAATAC | GTCGTCCAGCAGAGAATAGAAG |

# Supplementary Figure Legends

**Supplementary Figure 1. Identification of a variant in *MFSD3* causing ADNSHL of** **family HBZ02.**

**(A)** The pedigree of a six-generational family with segregating progressive ADNSHL. DNA samples were obtained from eight affected and six unaffected individuals (denoted by asterisks in red). Individuals who underwent whole-genome sequencing are marked with W in red. The hearing status of some family members was unknown (denoted by question mark in red). The patient V:1 (37 years old) exhibited only mild hearing loss at 8 kHz (30 dB HL) in the left ear, warranting close follow-up for monitoring potential auditory changes (denoted by question mark in green).

**(B)** Audiograms from selected individuals of family HBZ02. Threshold reveal the presence of mild to severe sensorineural hearing loss, which occurred at a late onset in all affected individuals. Younger individuals had less severe hearing loss compared with the older affected individuals, suggesting that hearing loss in the family is progressive in nature.

**(C)** Sanger sequencing showed that the *MFSD3* c.608C>T cosegregated with hearing loss. ADNSHL, autosomal-dominant, non-syndromic, sensorineural hearing loss; *MFSD3*, Major facilitator superfamily domain containing 3.

**Supplementary Figure 2. Expression patterns of *mfsd3* in zebrafish embryonic development.**

**(A)** qRT-PCR for twelve embryo development stages (0.2hpf, 1hpf, 2hpf, 3.7hpf, 6hpf, 24hpf, 30hpf, 48hpf, 72hpf, 96hpf, 120hpf and 144hpf) demonstrated different expression patterns of *mfsd3* during embryonic development (n=4).

**(B)** RNA-Seq for the *mfsd3* mRNA expression time course of zebrafish development across 18 time points from 1 cell to 120hpf exhibited a similar pattern to that observed in A (n=5). Error bars, SEM; hpf, hours post fertilization; **** P < 0.0001.

**Supplementary Figure 3. Phenotypes of *mfsd3* zebrafish morphants.**

**(A-C)** Observations on the general appearance of zebrafish embryos at 3-dpf. Compared with control MO, the *mfsd3* knockdown MO caused shorter body length, curved body axis and pericardial edema (B, C).

**(D)** In vivo imaging of live Brn3c: mGFP transgenic embryo at 3-dpf showed GFP expression from RGCs (retinal ganglion cells) and neuromasts (green dots) of the posterior lateral line and head. Zebrafish under control exhibited normal hair cells number **(E, F)**. Scale bar, 100μm; dpf, days post fertilization.

**Supplementary Figure 4. Expression levels of *Mfsd3* in mouse SGNs at different ages.**

Single-cell RNA-sequencing analysis depicting *Mfsd3* expression levels in SGNs of E14 (red), E18 (green), P3 (blue) and P21 (purple) mice. The raw sequencing data were obtained from NCBI’s Gene Expression Omnibus database under accession codes GSE165502, GSE117055 and GSE114997. SGN, spiral ganglion neuron.

**Supplementary Figure 5. Validation of anti-MFSD3 polyclonal antibody in HEK293T cells.**

HEK293T cells (provided by Vector Core, Chinese Institute for Brain Research, Beijing) were transiently transfected with pcDNA3.1-CMV-*MFSD3*-EGFP. The localization of EGFP-labeled MFSD3 (green) was not colocalized with anti-MFSD3(red). Scale bar, 5μm.
